# An investigation into the relationship between small intestinal fluid secretion and systemic arterial blood pressure in the anesthetized rat

**DOI:** 10.14814/phy2.12407

**Published:** 2015-05-27

**Authors:** Michael L Lucas, James D Morrison

**Affiliations:** School of Life Sciences, University of GlasgowWest Medical Building, Glasgow, G12 8QQ, U.K

**Keywords:** Absorption, blood pressure, jejunum, secretion, vasodilators

## Abstract

The effects of changes in the steady level of diastolic blood pressure on fluid flux across the jejunum has been investigated in the anesthetized rat during perfusion with a nutrient-free and Na^+^-free solution. Diastolic blood pressure was manipulated by intravenous infusions, during the jejunal perfusions, of vasodilators (vasoactive intestinal polypeptide, acetyl-*β*-methylcholine, and phentolamine) and a vasoconstrictor (arginine vasopressin), each of which acts through a different cellular mechanism. The outcome was that fluid flux was related by a parabolic relationship with diastolic blood pressure in which net secretion occurred over the range 40–100 mmHg, whereas net absorption was recorded at diastolic pressures exceeding 100 mmHg and below 40 mmHg. Against a background of normal absorption promoted by perfusion with 145 mmol L^−1^ Na^+^/5 mmol L^−1^ glucose solution, reductions in diastolic blood pressure markedly reduced the mean rate of fluid absorption by 58% overall, whereas the rate of glucose absorption remained unchanged. Our results were explained on the basis that vasodilatation led to increased capillary pressure and then to net filtration of fluid from the mesenteric capillary bed. Experiments in which *Escherichia coli* heat-stable toxin was added to the jejunal perfusate confirmed the absence of a secretory response, which was consistent with the absence of effect of the toxin on diastolic blood pressure.

## Introduction

The prevailing view of small intestinal secretion has been that the crypts of Lieberkühn secrete a solution of Na^+^, Cl^−^, and 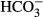, whereas absorption occurs at the villi, thus forming an *external fluid circuit* (Gregory 1962). This separation of function has received considerable support from experiments on intestinal mucosa in which selective inactivation of the crypts did not impair the absorptive function of the villi, whereas inactivation of the villi was without effect on fluid secretion by the crypts (Serebro et al. 1969; Roggin et al. 1972). The processes underlying crypt cell secretion were subsequently investigated by Huott et al. (1988) who showed that, as a consequence of low intracellular Na^+^ through the action of the basolateral Na^+^/K^+^ ATPase, Na^+^ entry drives the basolateral Na^+^/K^+^/2Cl^−^ cotransporter to effect K^+^ and Cl^−^ entry. K^+^ ion is recycled through the basolateral K^+^ conductance, whereas the elevated intracellular Cl^−^ concentration causes the efflux of Cl^−^ ion through the luminal CFTR (cystic fibrosis transmembrane conductance regulator) into the lumen of the crypt. Cl^−^ ion flux is thus regarded as the driving force for the accompanying secretion of Na^+^ and water (Barrett and Keely 2000). However, the preparation used by Huott et al. (1988) which consisted of monolayers derived from metastatic colonic carcinoma cells may not be entirely representative of normal function as perfused colonic crypts have been shown to be absorptive in function rather than secretory and that secretions were evoked only on stimulation by an agonist (Singh et al. 1995). Overall, the small intestine in the basal state shows net absorption as indicated by the positive mucosal to serosal flux of Na^+^ and Cl^−^ ion (Love 1969; Field et al. 1972; Guandalini et al. 1982). However, the extent to which agonists can reverse this absorption into net secretion is important as it underpins the understanding of the debilitating secretory diarrhea caused by toxins from *Vibrio cholerae* and *Escherichia coli*. The latter produces two main toxins: a heat labile toxin producing cholera-like responses and a heat-stable toxin which has been the subject of intensive investigation (Sach 1975). The fluid loss into the lumen associated with these infections is said to be driven primarily by increased Cl^−^ secretion through the CFTR, which is then followed by Na+ and water secretion. With respect to *V*. *cholerae*, the CFTR is activated by increased levels of cAMP produced by stimulation of adenylate cyclase by cholera toxin (Chen et al. 1971; Field et al. 1972; Burleigh & Borman, 1997), whereas *E*. *coli* heat-stable toxin acts by increasing cGMP levels through activation of guanylate cyclase (Field et al. 1978; Giannella and Drake 1979; Rao et al. 1981; Guandalini et al. 1982). Even though both toxins cause considerable fluid loss from the intestine of infected humans (Sach 2011), inconsistencies have arisen in the results from experimental studies on animal models. Net Cl^−^ secretion which is associated with increased negativity of the transmucosal short circuit current (I*sc*) has been demonstrated after luminal exposure to *V*. *cholerae* toxin both in vitro and in vivo (Love 1969; Field et al. 1972; Krejs et al. 1978) though, by contrast, this was not shown by Norris et al. (1967) who reported that I*sc* remained constant after 6 h of choleragen-induced fluid secretion in vivo nor by Moritz et al. (1972) who showed an absence of net Cl^−^ despite marked fluid secretion. For *E*. *coli* toxin-induced fluid loss, the position is also unclear as the basal Cl^−^ flux in the absorptive direction does fall to zero after exposure to toxin but does not reverse into net secretion (Field et al. 1978; Rao et al. 1981; Vaandrager et al. 2000), though Guandalini et al. (1982) have reported a full reversal from net absorption to net Cl^−^ secretion. The time courses of action also differ very markedly. *E*. *coli* toxin acts remarkably rapidly (Evans et al. 1973; Field et al. 1978; Giannella and Drake 1979; Guandalini et al. 1982; Huott et al. 1988), whereas *V*. *cholerae* toxin has a much slower time-course of action extending into hours (Field et al. 1972; Evans et al. 1973; Carey and Cooke 1986; Petritsch et al. 1992; Burleigh and Borman 1997), which has been explained by the delayed penetration of the toxin into the mucosal cells (Burleigh and Borman 1997). A marked lack of consistency also becomes apparent when relating the results obtained in vitro to those obtained in vivo. Experimental studies in both humans and animals have shown that *V*. *cholerae* toxin consistently caused copious intestinal secretions (Benyajati 1966; Norris et al. 1967; Carpenter et al. 1969; Love 1969; Moritz et al. 1972; Evans et al. 1973; Sach et al. 1976; Krejs et al. 1978; Petritsch et al. 1992). By contrast, the position regarding *E*. *coli* heat-stable toxin is less clear. There are instances in which net secretion has been reported (Evans et al. 1973; Vaandrager et al. 2000) though, in other studies, no significant net secretion was recorded (Sach et al. 1976; Rolfe and Levin 1994; Lucas et al. 2005, 2008, 2011). As there are disparities in terms of Cl^−^ ion secretion, latency of action and the amount of net fluid secretion between two major effectors of secretory diarrhea, *viz*. *V*. *cholerae* and *E*. *coli* toxins, there are compelling reasons to investigate whether there are other mechanisms aside from Cl^−^ ion secretion which promote intestinal fluid secretion.

Our approach to this problem has involved a consideration of mucosal blood pressure, which stems from the observation of Claude Bernard in 1859 (in Gregory 1962), and confirmed by Gregory (1947), that denervation of the small intestine in vivo leads to copious intestinal secretions (*paralytic secretion*). As denervation, particularly extirpation of the coeliac ganglion as in the case of Claude Bernard, must inevitably affect the pressure within the mucosal vasculature, we have sought to investigate the extent to which intestinal fluid movement is affected by the prevailing arterial blood pressure (ABP) in conditions in which intestinal absorption was minimized by use of a Na^+^-free and nutrient-free perfusate. Manipulation of the peripheral resistance of the vasculature was achieved by intravenous (I.V.) infusions of vasoactive agents over the duration of the perfusion of the jejunum. A depressor action through vasodilatation was mediated with (1) vasoactive intestinal polypeptide (VIP), the neurotransmitter form of secretin, which through receptor binding activates adenylate cyclase to cause cAMP production, activation of PKA and phosphorylation of myosin light chain kinase (Fölsch et al. 1980; Kamm and Stull 1985), (2) acetyl-*β*-methylcholine (MC), a stable analog of acetylcholine, which binds to muscarinic receptors to activate phospholipase C to cause an elevation of intracellular IP_3_ and intracellular Ca^2+^, subsequent activation of nitric oxide synthase leading to NO production (Eglen 2005), and (3) phentolamine which blocks *α*-adrenoceptors preventing the pressor action of catecholamines, which are mediated through elevation of intracellular IP_3_ and intracellular Ca^2+^ (Bylund et al. 1994). A pressor action was mediated by infusion of arginine vasopressin (AVP) acting on V_1_ receptors causing an elevation of intracellular IP_3_ and intracellular Ca^2+^ to cause smooth muscle contraction (Thibonnier et al. 2001). The outcome of these experiments was that there was clear evidence of intestinal secretion, but only when the diastolic blood pressure (DBP) fell within the range 40–100 mmHg with absorption occurring above and below this range. As a possible explanation for this result is that vasodilatation leads to increased mucosal capillary pressure which, in turn, leads to increased capillary filtration of fluid and hence secretion, we have also sought to investigate its plausibility by devising a pressure/flow model using established cardiovascular values.

## Methods

Experiments were carried out on male rats of 250–400 g in accordance with the Animals (Scientific Procedures) Act 1986. The animals were fasted overnight but were allowed a 5% sucrose drink in addition to water. They were anesthetized with an intraperitoneal (I.P.) injection of pentobarbitone sodium (Rhône Mérieux, Harlow, U.K.) at a dose of 80 mg kg^−1^. The criterion for anesthesia was abolition of the hind limb flexor withdrawal reflex. In order to maintain the level of anesthesia, supplementary I.P. injections (24 mg kg^−1^) were given as required. At the end of the experiments, the animals were killed with an I.V. injection of 100 mg Euthatal (Merial Animal Health, Harlow, UK).

### Surgical procedures

The surgical procedures consisted of the following: tracheostomy to allow tracheal aspiration and artificial ventilation if necessary, cannulation of the carotid artery to monitor the ABP, cannulation of the external jugular vein and occasionally the external iliac vein for slow infusions (Portex cannulae of outer diameter 1.0 mm and containing heparinized saline at 100 I.U. heparin mL^−1^, Leo Laboratories Ltd., Princes Risborough, UK) and bilateral vagotomy to abolish vago-vagal reflexes. Following a midline incision of the abdominal wall, the pyloroduodenal junction was ligated to prevent entry of gastric secretions into the duodenum and the small intestine was exposed. Cannulae of 4.0 mm outer diameter were inserted and tied in at both ends of a 30 cm length of jejunum located distal to the ligament of Treitz. The animal's temperature was maintained as near as possible to 37°C and at the end of the experiment, the length of the perfused jejunal loop was measured and a sample taken for histology.

### Experimental procedure

The warmed perfusate was circulated by a Watson–Marlow peristaltic pump at a rate of 1.0 mL min^−1^ from a 50 mL measuring cylinder standing in a heated water bath through polythene tubing of internal diameter 3.0 mm into the jejunal loop in situ from which the effluent was then returned by another length of tubing to the measuring cylinder. First, the jejunum was flushed through with 25 mL of 154 mmol L^−1^ choline chloride solution (Sigma C1879; Sigma, Poole, UK) and the perfusate discarded. The experimental perfusate consisted of 40.0 mL 154 mmol L^−1^ choline chloride solution containing 100 *μ*mol L^−1^ EIPA (5-(N-Ethyl-N-isopropyl) amiloride: Sigma A3085; Sigma) (Na^+^/H^+^ antiport antagonist) at 37°C and pH 7.0, which was recirculated from the measuring cylinder standing in the water bath for a timed period of at least 1 h after which the perfused fluid was then recovered from the jejunal loop and any change in the amount of perfusate was determined as the difference in the weight of fluid before and after perfusion. Steady reductions in peripheral resistance, indicated by the systemic blood pressure recordings, were achieved by slow I.V. infusions over 45–60 min using a micrometer-driven syringe of either 10–50 *μ*g VIP (Sigma V6130; Sigma) or 100–300 *μ*g acetyl-*β*-methylcholine bromide (Sigma A2126; Sigma) dissolved in 1.5 mL isotonic saline, whereas 200–500 *μ*g phentolamine mesylate (Rogitine, Ciba, Basel, Switzerland) was injected by slow bolus injection in 0.2 mL isotonic saline. Increases in peripheral resistance were achieved by slow I.V. infusions of 2.5–8.0 *μ*g arginine vasopressin (Pitressin, Parke-Davis, Pontypool, UK) also dissolved in 1.5 mL isotonic saline. In addition, further experiments were undertaken involving the perfusion of the jejunum with Krebs-phosphate solution containing 145 mmol L^−1^ Na^+^ and 5.0 mmol L^−1^ glucose pH 7.4 in order to promote absorption. In one set of experiments, the effects of the vasodilator agents I.V. on fluid absorption, measured by the method described above, and on glucose absorption, measured in duplicate with the Accu-Chek glucose meter (Roche Diagnostics, Burgess Hill, UK), were determined. In the second set of experiments, the effect of the addition of 1.0 *μ*g *E*. *coli* heat-stable toxin (STa) (Sigma E-5763; Sigma) to the luminal perfusate was tested.

### Histology

After completion of the experiments, a 5 cm length of the perfused jejunum was infused with 10% formal saline and immersed in 10% formal saline overnight and then processed for paraffin embedding. Transverse sections were made from several locations in each segment of gut and stained with hematoxylin/eosin and alcian blue for light microscopy.

### Data analysis

Application of the appropriate statistical tests was made with Minitab 15 (Minitab UK, Coventry, UK). Comparisons against zero were made with the *t*-test, comparisons of changes in ABP and heart rate were made with the paired *t*-test and the coefficient of determination (*R*^2^) was obtained by regression analysis, with statistical significance taken as *P* < 0.05. The results throughout are expressed as the mean ± SEM.

## Results

### Arterial blood pressure

**Figure 1:**
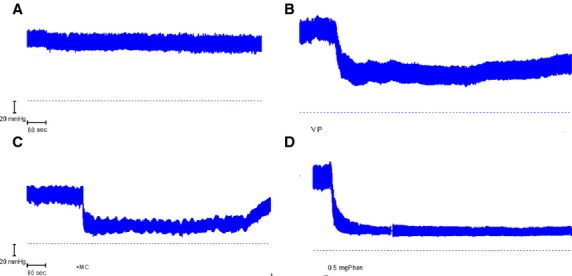
Arterial blood pressure traces from four different animals during jejunal perfusions with 154 mmol L^−1^ choline chloride and EIPA showing: (A) Control recording without I.V. infusion. (B) Slow infusion of 50 *μ*g VIP I.V. (VIP). (C) Slow infusion of 300 *μ*g acetyl-*β*-methylcholine I.V. (MC). (D) I.V. injection of 0.5 mg Phentolamine (Phen). The dashed horizontal line denotes 0 mmHg. The disturbance in trace D was caused by flushing of the arterial cannula.

The results were obtained from a total of 39 rats in which the ABP at the commencement of the experiments had the mean values of 132/104 mmHg with a mean ABP of 114 *±* 2.0 mmHg, a pulse pressure of 28 *±* 1.0 mmHg and a heart rate of 388 *±* 5 bpm (beats per minute). While many experiments ended with the infusion of a vasoactive agent, in nine animals a second control perfusion was undertaken. In these cases, after 4–5 h, the mean ABP of 116 *±* 3.6 mmHg and the pulse pressure of 27 *±* 2.1 mmHg were unchanged compared with the starting values (*P* = 0.41). There were also no adverse effects of perfusion of the jejunum with 154 mmol L^−1^ choline chloride and 100 *μ*mol L^−1^ EIPA when these were perfused as an initial control procedure in 10 animals (Fig.1A): the DBP after perfusion for 1 h (110 *±* 3.7 mmHg) was not significantly different from that at commencement of perfusion (113 *±* 5.1 mmHg) (*P* = 0.40).

During the jejunal infusions, the diastolic blood pressures through I.V. infusion of vasoactive agents ranged from 128 mmHg during AVP infusion down to as low as 20 mmHg with continuous I.V infusion of VIP and MC and with a single bolus injection of phentolamine which caused sustained reductions (Fig.1B–D). Initially, MC caused a transient reduction in heart rate to 30–50% of the control value with an associated increase in pulse pressure, both of which rapidly subsided, presumably due to desensitization of the cardiac muscarinic receptors. The effects of the vasoactive agents on cardiac function during the period of I.V. infusion were assessed after the DBP had reached a steady state. Compared with the control value for each animal, neither MC nor phentolamine significantly affected heart rate or pulse pressure (*P* > 0.20). The pulse pressure remained unchanged with VIP (*P* = 0.64) though there was a small, but significant reduction in heart rate by 26 *±* 7 bpm (6%) (*P* = 0.006). While these results indicate a degree of constancy of cardiac function, this was not the case with AVP which significantly increased pulse pressure by 26 *±* 7.0 mmHg (100%) (*P* *<* 0.001) and was associated with a significant reduction in heart rate by 58 *±* 7 bpm (15%) (*P* *<* 0.01).

### Jejunal fluid flux

**Figure 2:**
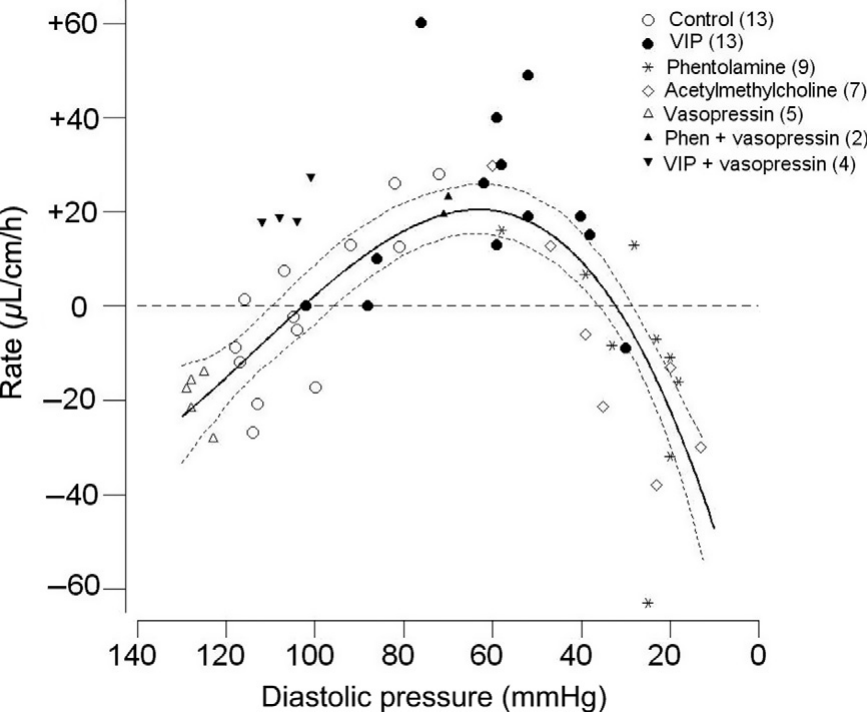
Magnitude of rate of secretion (positive values) and absorption (negative values) for the perfused jejunal loop in vivo at different prevailing levels of diastolic blood pressure caused by vasoactive agents I.V. denoted by the different symbols. The numbers in parentheses show the number of observations. The continuous curve follows the equation *y* = 0.00012*x*^3^ − 0.040*x*^2^ + 3.7*x –* 80, whereas the dashed curves show the upper and lower 95% Confidence Interval for the regression curve.

For the 13 control perfusions, the amount of fluid secreted (denoted +) or absorbed (denoted −) by the jejunal loop did not seem, at first sight, to follow a consistent pattern with a nonsignificant mean value of −0.4 *±* 4.8 *μ*L cm^−1^ h^−1^ (*P* = 0.94). However, further analysis showed a significant inverse relationship between net fluid flux and the DBP (*R*^2^ = 65%*, P*_slope_ = 0.001), such that net secretion was recorded for blood pressures of <100 mmHg and net absorption was recorded for blood pressures of >100 mmHg (Fig.2, open circles). The outcome was the same when the mean ABP was used in place of DBP.

When the results for the four vasoactive agents were included, now giving a total of 52 results, a definite pattern emerged in the form of a highly significant paraboloid relationship between flux rate and DBP (*R*^2^ = 72%*, P* *<* 0.001) (Fig.2). Over the range of DBP of 135 to 100 mmHg (AVP and control results), net absorption was recorded, whereas over the range 100 to 40 mmHg (VIP, MC, phentolamine, and some control results), net secretion as high as +60 *μ*L cm^−1^ h^−1^ occurred. Finally, at DBP of <40 mmHg (MC and phentolamine), absorption of increasing magnitude was recorded. A similar relationship with that shown in Figure2 was obtained when the rate of fluid flux was related to the mean ABP (*R*^2^ = 66%*, P* *<* 0.001).

### Coinfusion of vasoactive agents

**Figure 3:**
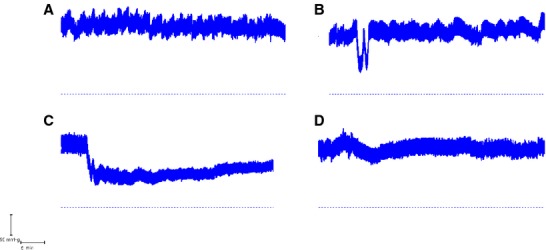
Arterial blood pressure traces from a sequence of procedures in the same animal during jejunal perfusions with 154 mmol L^−1^ choline chloride and EIPA: (A) Control recording without I.V. infusion with an absorption rate of −8.8 *μ*L cm^−1^ h^−1^ at a DBP of 118 mmHg. (B) Slow coinfusion of 50 *μ*g VIP I.V. and 3 *μ*g AVP I.V. with a secretion rate of +17.8 *μ*L cm^−1^ h^−1^ at a DBP of 112 mmHg. (C) Slow infusion of 50 *μ*g VIP I.V. with secretion rate of +26.0 *μ*L cm^−1^ h^−1^ at a DBP of 62 mmHg. D. Control recording without I.V. infusion with an absorption rate of −5.0 *μ*L cm^−1^ h^−1^ at a DBP of 104 mmHg. In B, the VIP infusion caused an initial dip in blood pressure which was restored to normal with the commencement of AVP infusion. In C, the marked fall in blood pressure marks the start of VIP infusion. The dashed horizontal line denotes 0 mmHg.

**Figure 4:**
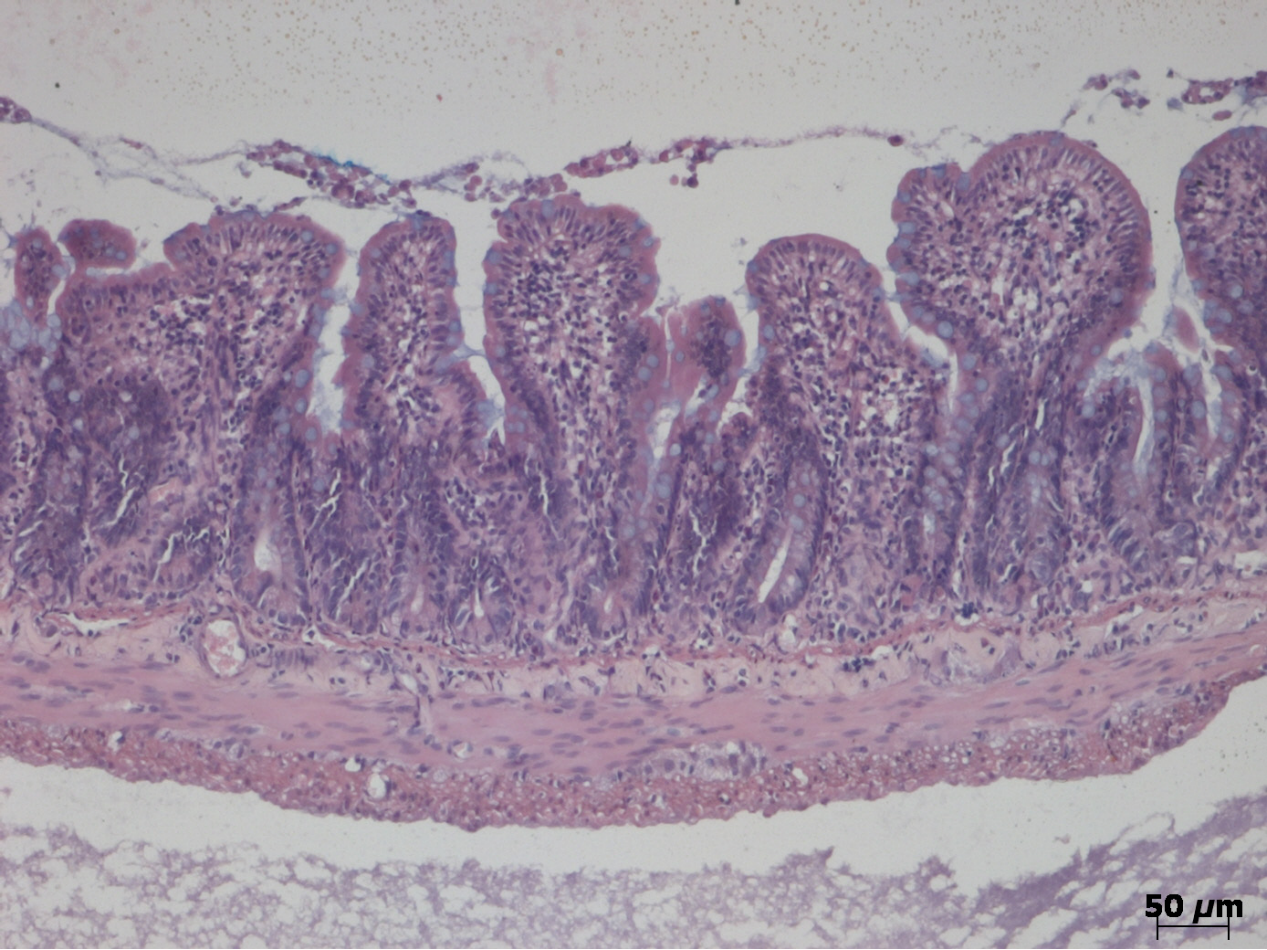
Transverse section of jejunum with lumen at the top after jejunal perfusion experiment with 154 mmol L^−1^ choline chloride and EIPA in which I.V. bolus injection of 1 mg phentolamine reduced the DBP to 18 mmHg for 1 h. This was followed by slow infusion of 10 *μ*g AVP to restore the DBP to 70 mmHg for 1 h. Hematoxylin, eosin and alcian blue staining.

The basis of the coinfusion experiments was to use infusion of AVP into the jugular vein to nullify the action of the vasodilator infused into the external iliac vein in order to maintain a steady ABP. When infused alone, AVP elevated the DBP to 127 *±* 1.1 mmHg which was associated with a significant net absorption rate of −20 *±* 2.5 *μ*L cm^−1^ h^−1^ (*P* = 0.001) (5 experiments). By contrast, VIP infusion (13 experiments) resulted in a significant fall in DBP to 62 *±* 5.8 mmHg which was associated with net secretion at a significant rate of +20.9 *±* 5.5 *μ*L cm^−1^ h^−1^ (*P* = 0.003). When AVP and VIP were coinfused in four experiments (Fig. 3), the DBP was maintained at 106 *±* 2.4 mmHg, whereas the secretion rate, instead of being zero as might have been expected from Figure2, was significantly elevated at +20.6 *±* 2.33 *μ*L cm^−1^ h^−1^ (*P* = 0.003) (Fig.2, inverted solid triangles). These results thus indicate that VIP still caused net secretion when the ABP was maintained at a normal value. In two further experiments to test the tolerance to low DBP, AVP was infused subsequent to an injection of phentolamine which had reduced the DBP to 25 mmHg and had caused a mean absorption rate of −12 *μ*L cm^−1^ h^−1^. In both cases, AVP restored the DBP to 70 mmHg and absorption was transformed into secretion at a mean rate of +21 *μ*L cm^−1^ h^−1^. This indicated that secretion could be restored even after the jejunum had been subjected to an extended period of low DBP. This was further supported by the normal histological appearance of the mucosa under light microscopy as shown in Figure4. Routine histology confirmed the integrity of the jejunal mucosa in all experimental preparations.

### Effects of reduced DBP on normal absorption

**Figure 5:**
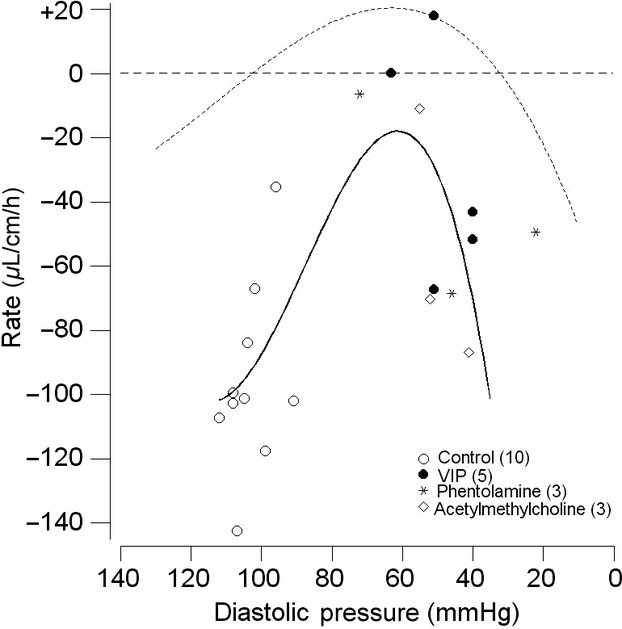
Magnitude of rate of secretion (positive values) and absorption (negative values) for the jejunal loop in vivo perfused with 145 mmol L^−1^ Na^+^ and 5.0 mmol L^−1^ glucose solution at different prevailing levels of diastolic blood pressure caused by vasoactive agents I.V. denoted by the different symbols. The numbers in parentheses show the number of observations: some experiments contained two test infusions. The continuous solid curve follows the equation *y* = 0.0011*x*^3^ − 0.30*x*^2^ + 24*x –* 623, whereas the dashed curve is reproduced from Figure2.

The effects of reduced DBP produced by I.V. infusion of VIP, MC or phentolamine on normal absorption, which was enabled by perfusion of the jejunum with 150 mmol L^−1^ Na^+^/5.0 mmol L^−1^ glucose Krebs solution, was determined in nine animals. Each jejunal perfusion at a reduced DBP was paired with a perfusion at normal DBP and the results are summarized in Table1. There was, in every case, a marked reduction in the rate of absorption from the control value (Fig.5, open circles) to the test value when the DBP was reduced (Fig.5, special symbols). The rate of absorption and DBP showed a significant parabolic relationship, shown by the solid curve, similar to that in Figure2, shown by the dashed curve, though with downward shift and a reduced goodness of fit (*R*^2^ = 53%*, P* = 0.004). Overall, the mean control rate of −97 *μ*L cm^−1^ h^−1^ was significantly reduced by 58% to −41 *μ*L cm^−1^ h^−1^ when the DBP was reduced (*P* *<* 0.001) (Table1). Of special note was that the rate of glucose absorption remained constant when the DBP was reduced (*P* = 0.78).

**Table 1:** Effects of reduced diastolic blood pressure on normal absorption.

	Normal	Reduced DBP	*P*
Diastolic blood pressure (mmHg)	106 ± 1.4	50 ± 3.9	*P* < 0.001
Fluid flux (*μ*L cm^−1^ h^−1^)	−97.2 ± 7.6	−41.3 ± 9.7	*P* < 0.001
Glucose absorption (*μ*mol cm^−1^ h^−1^)	−1.6 ± 0.14	−1.7 ± 0.32	*P* = 0.78

These results thus demonstrate the effectiveness of reduced DBP in markedly reducing the rate of fluid absorption in the presence of normal glucose absorption.

## Discussion

In this study, it has been shown for the perfused jejunal loop in vivo that, when the conditions for absorption are removed *viz*. absence of glucose and Na^+^ (which was replaced by the impermeant ion choline) and inhibition of the Na^+^/H^+^ antiport by EIPA, net secretion was possible with rates as high as +60 *μ*L cm^−1^ h^−1^. This net secretion, however, very much depended on the prevailing ABP such that net absorption occurred at high and low DBPs, whereas net secretion occurred in the range between 40 and 100 mmHg for the DBP (Fig.2). So, consequently, secretion could occur in the control condition, provided that the DBP erred toward lower values. Small but significant levels of secretion have also been recorded with choline chloride perfusion by Lee (1977) and Lucas et al. (2005), though these cannot be related to the ABP values which were not documented in these studies.

In order to achieve an extended range of DBP values, intervention was required in the form of I.V. infusion of vasoactive agents. The three vasodilators which were used (VIP, MC, and phentolamine) and the vasoconstrictor AVP each acts through a different cellular mechanism (Introduction). Given that phentolamine has its actions through its antagonism of the sympathetic neurotransmitter noradrenaline, all four vasoactive agents have been shown to act on the mesenteric vasculature, including the villous arcade, predominantly through actions on the arterial vasculature thus affecting inflow into the mesenteric capillary beds (Dresel and Wallentin 1966; Krejs et al. 1978; Hollinger et al. 1983; Tesfamariam and Halpern 1987; Warner 1990). As shown by Figure2, net jejunal secretion was not associated with a specific vasoactive agent but, rather, was dependent upon the DBP caused by that vasoactive agent so that secretion occurred over the range from 100 mmHg down to 40 mmHg. By contrast, DBP values above 100 mmHg and below 40 mmHg were associated with net absorption of fluid. An important consideration is the reliance which can be placed upon the results obtained from our experiments. As confirmation of the animal's physiological condition, in those experiments which ended with a control perfusion, the animals still maintained a normal ABP after 4–5 h experimentation. Additionally, perfusion of the jejunum with 154 mmol L^−1^ choline chloride was without an adverse effect *per se* on the DBP, and histological examination of the jejunum after each experiment showed normal mucosal structure under light microscopy. The robust nature of the results was additionally confirmed by interleaving different interventions in the same animal. So, for example, two periods of net secretion during VIP infusion were then followed by net absorption when the DBP was allowed, on cessation of the VIP infusion, to return to its control value (Fig.3 and legend). The converse result was also obtained when a period of net absorption at reduced DBP values was then followed by net secretion when the DBP was subsequently increased to a new steady level. In these cases, the jejunal mucosa still showed a normal appearance under light microscopy (Fig.4). Throughout, we saw no indications of mucosal cell shedding, as reported by Lee (1977) during exposure to secretagogues, which is in accord with the normal appearance of the ileum reported by Patnaik and Ghosh (1966) after cholera-induced secretion.

While the results in Figure2 indicate DAB to be a factor responsible for secretion, the possibility of a direct secretagogue action of the agonists, possibly on the crypt cells, needs to be examined as such a stimulatory action has been claimed for both MC (Isaacs et al. 1976; Singh et al. 1995) and VIP (Barbezat and Grossman 1971; Krejs et al. 1978; Singh et al. 1995; Banks et al. 2005). In the present study, MC caused secretion in only two experiments, whereas absorption occurred in five experiments, which is not what would be expected if MC were stimulating the crypt cells directly. Even with our limited subset of seven data points, fluid flux was highly significantly inversely related to DBP (*R*^2^ = 80%*, P*_slope_ = 0.002), indicating that DBP was the determining factor. The results for VIP infusion which, overall, caused appreciable net secretion were the subject of further investigation. When, while VIP was being infused I.V., simultaneous infusion of AVP was undertaken to neutralize the anticipated fall in ABP (Fig.3B), an appreciable level of secretion of +20.6 *±* 2.33 *μ*L cm^−1^ h^−1^ (*P* = 0.003) still occurred (Fig.2, solid triangles). This may indicate an ABP-independent action of VIP in the form of an effect on the crypts as suggested by results from the Thiry-Vella loop preparation in conscious dogs (Barbezat and Grossman 1971), perfused jejunal loops in anesthetized dogs (Krejs et al. 1978) and perfused colonic crypts (Singh et al. 1995). However, it would also be consistent with elevated capillary pressure despite normal ABP or an increase in the capillary filtration coefficient.

The reason for the dependence of fluid flux across the jejunum on ABP may stem from the consequences that the prevailing systemic ABP has on the blood flow in the mucosal capillary bed. In the normal state, the major fall in pressure occurs across the arterioles so that blood enters the capillary bed at a relatively low nonpulsatile pressure. At this point, due to the high permeability of the capillary vessel walls, the Starling forces have their action so that the hydrostatic pressure drives fluid out of the capillaries and the plasma protein oncotic pressure draws fluid into the capillaries. The traditional view is that, at the arteriolar end of the capillary bed, the excess of hydrostatic pressure over oncotic pressure results in net filtration out of the capillaries into the interstitial fluid, whereas at the venular end of the capillary bed, the hydrostatic pressure falls to below that of the oncotic pressure, so that absorption of fluid occurs. However, this does not take into account the interstitial tissue pressure which is slightly subatmospheric with the result that, in a well perfused capillary bed, filtration occurs along the entire length of the capillary bed with the exuded fluid drained away by the lymphatic system (Levick 2003). This is the situation which may be expected when the DBP is positioned at 100 mmHg (Fig.2). When there is systemic vasoconstriction so that the DBP is in excess of 100 mmHg, as caused by AVP, this will lead to reduced perfusion of the capillary bed and a reduction in the hydrostatic pressure so that net absorption will arise from the interstitial fluid which will, in turn, lead to absorption through the mucosa from the lumen. Such a reduction in pressure has been reported in mesenteric first-order arterioles by Bohlen and Gore (1979) in response to sympathetic nerve stimulation. The same outcome, *viz*. net absorption, will also occur at DBP below 30–40 mmHg but for a different reason: due to the severe reduction in ABP resulting from the widespread vasodilatation caused by the vasoactive agent, the blood flow through the capillary bed will be reduced to such an extent that the hydrostatic pressure now falls so that net absorption again occurs. This effect of stasis of the blood flow on capillary hydrostatic pressure has been measured directly in frog mesenteric capillaries by Landis (1926).

By contrast, over the range 40–100 mmHg, when the vasodilatation is more modest, this would have the effect of reducing the velocity of the blood flow in the mucosal capillary bed with the consequence that the hydrostatic pressure will increase. This is in keeping with Bernouilli's principle (Levick 2003) and is confirmed by direct measurements in mesenteric capillaries in which dilatation led to elevation of the pressure within individual capillaries in frog by 3–4.5 mmHg (Landis 1927), while a 50% reduction in arterial blood pressure resulted in a mean increase in capillary pressure of 14 mmHg in cat (Königes and Ottó 1937). As a consequence of the increased hydrostatic pressure in the capillary bed, this will lead to increased filtration of fluid from the capillary bed into the interstitial fluid space within the villi and, then, through the mucosal epithelium into the lumen. At a DBP of 40 mmHg, the pressure into the capillary bed is now so reduced that the capillary pressure declines passively with arterial pressure. The consequence is that outward filtration is countered by absorption due to the capillary oncotic pressure so that zero net flux results at this pressure. With the simplification of setting the hydrostatic pressure and the oncotic pressure in the intestinal lumen to zero, the main force for driving fluid into the lumen will be the difference between capillary hydrostatic and capillary oncotic pressure (see Appendix 1). As the oncotic pressure is essentially constant, the dominant factor for filtration is thus the hydrostatic pressure and it is this which increases when the DBP is reduced. The route for the exuded fluid through the mucosa has been attributed to passage through the protein network of the tight junctions located between adjoining enterocytes (DiBona et al. 1974; Anderson and Van Itallie 1995), though passage through aquaporins (AQP6) which are concentrated in the enterocytes at the villus tips also needs to be considered, though with the qualification that AQP6 appears to be pH sensitive (Laforenza et al. 2009). With respect to the proposed process of filtration through the intestinal mucosa, there are previous studies in which venous pressure has been increased and the plasma oncotic pressure has been reduced. In both cases, the normal absorptive function of the small intestine was reversed into fluid secretion due to increased filtration from the capillary bed (Lee 1973; Yablonski and Lifson 1976). There are also well documented precedents of other epithelia which secrete through ultrafiltration *viz*. the choroid plexus which secretes cerebrospinal fluid (Pollay et al. 1983) and the ciliary epithelium of the eye which secretes 20% of the aqueous humor production by this means (Davson 1990).

The rate of fluid absorption during perfusion with Krebs solution containing Na^+^ and glucose occurred at the high value of −97 *μ*L cm^−1^ h^−1^ which is similar to previously reported values for the rat (Humphreys and Earley 1971; Lee 1973; Lucas et al. 2008; Morrison 2012). When the DBP was reduced from its normal value of 106 mmHg down to a range extending from 72 to 22 mmHg, there was a dramatic reduction in the mean rate of fluid absorption by 58% overall (Fig.5, Table1). That this occurred while the rate of glucose absorption remained unchanged indicated that the effect of the reduced DBP occurred during the normal absorptive process, which is consistent with the results of Serebro et al. (1968) who showed that glucose absorption remained constant during cholera-stimulated fluid secretion. If the mean reduction in the rate of absorption by 56 *μ*L cm^−1^ h^−1^ (Table1) is taken to be the rate of fluid secretion which is countering normal fluid absorption, this amounts to three times the peak level of fluid secretion apparent with the Na^+^-free/glucose-free perfusate (Fig.2). As glucose absorption must be accompanied by Na^+^ absorption which, in turn, is followed by greater fluid absorption (Schultz et al. 1974), a possible explanation for the inferred higher rate of fluid secretion is that the absorptive process is now providing more fluid for the secretory process. While the secretion described with Na^+^-free and glucose-free perfusate may be considered to replicate the fasted gut, even in the presence of Na^+^ and glucose such as is used in oral rehydration therapy, there would still be a substantial action of reduced DBP in reducing the rate of fluid absorption. It was also noted that VIP infusion produced the greatest reductions in the rate of absorption, reducing it to zero in one case and driving it into active secretion in a second case (Fig.5). This action of VIP through reducing ABP and possibly increasing capillary pressure and permeability may contribute substantially to the severe secretory diarrhea of pancreatic cholera (Verner–Morrison syndrome) in which VIPoma cells in the Islets secrete excessive quantities of VIP into the circulation (Mansour and Chen 2004).

### Formulation of a model

In order to test numerically the plausibility of our proposal that reduced diastolic blood pressure results in increased capillary pressure which then leads to fluid exudation and secretion into the intestinal lumen, we have formulated a model which is based on established cardiovascular values and on a number of simplifying assumptions. The capillary vessels are assumed to be of a constant number, of a constant length and of a uniform diameter and, when the diameter changes, it does so uniformly across the capillary bed. It is assumed that the viscosity of the blood remains constant, and that the cardiac output remains constant during infusion of the vasoactive agents. The latter is not unreasonable given that, with the exception of AVP, heart rate and pulse pressure remained essentially unchanged during the I.V. infusions.

**Figure 6:**
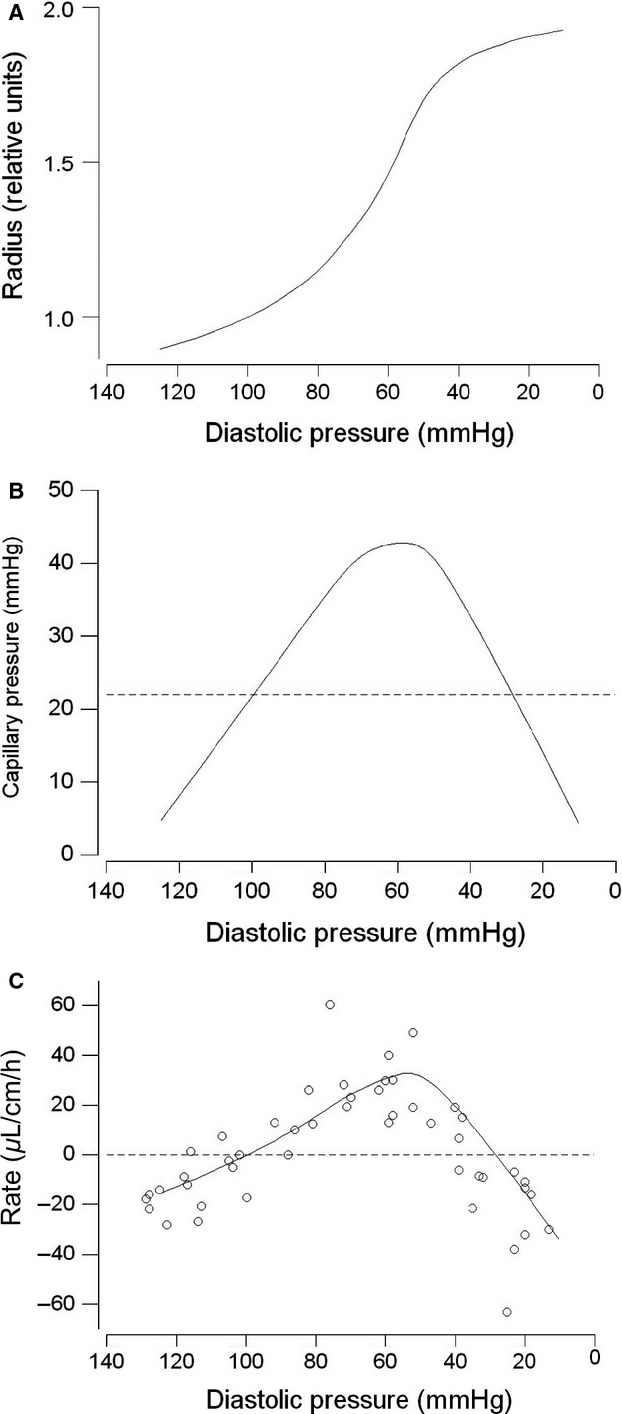
(A) Relative values allocated for arteriolar radius against diastolic pressure referenced against a value of 1.00 at 100 mmHg. (B) Calculated values of capillary pressure at different diastolic pressure values using the radius values in A with the horizontal dashed line showing 22 mmHg. (C) Calculated values for net fluid flux across the mesenteric capillary bed in response to reducing levels of diastolic blood pressure (continuous curve), showing data points from Figure2 as open circles but excludes coinfusions of VIP and AVP. Further details are given in the text.

From the relationship 

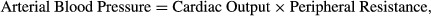
the peripheral resistance into the arteriolar end of the mesenteric capillary bed was calculated as: 

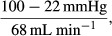
where 100 mmHg is the DBP (Wheeler et al. 2002; McHarg et al. 2004), 22 mmHg is the pressure at the arteriolar end of the mesenteric bed (Landis and Pappenheimer 1963), and 68 mL min^−1^ is the cardiac output (Slama et al. 2003). This gives a value for the peripheral resistance of 1.15 units. Then, the value for the blood vessel radius (*r*) expressed in relative units was incremented from 0.90 at DBP = 125 mmHg to 1.92 at DBP = 10 mmHg (as shown in Fig.6A). These values are well within the range recorded from mesenteric arterial branches after denervation or during various levels of transmural stimulation of sympathetic nerves (Bohlen and Gore 1977; Tesfamariam and Halpern 1987).

Then, using *r*^−4^ as a simplification of Poiseuille's expression for resistance, the hydrostatic pressure at the arteriolar end of the mesenteric capillary bed was obtained from: 

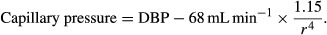


The form of the resulting relationship was that the capillary pressure increased almost linearly from DBP = 125 mmHg to a peak value of 42 mmHg at DBP = 60 mmHg after which it declined linearly as the DBP continued to fall (Fig.6B). Transformation of the hydrostatic pressure into net fluid flux was made on the following basis. In the rat mesenteric bed, the hydrostatic pressure at the arteriolar end has been reported to be 22 mmHg and 12.5 mmHg at the venous end, whereas the oncotic pressure varies between 16 and 21 mmHg along the length of the capillary bed (Landis and Pappenheimer 1963). It was assumed that, at an arteriolar pressure of 22 mmHg, the Starling forces are in balance and no net fluid flux occurs. As the excess between this value and the peak capillary pressure value of 42 mmHg (Fig.6B) leads to exudation of fluid at a projected rate of +20 *μ*L cm^−1^ h^−1^ (Fig.2), this gives a filtration constant of 1.0 *μ*L cm^−1^ h^−1^ mmHg^−1^ which then must be multiplied by a factor for the changing surface area (2*πrl*) which is taken to be proportional to *r*, thus giving a filtration coefficient of 1.0 *r*. The outcome for the projected rate of fluid secretion/absorption is shown in Figure6C; so, at high and low DBP, there is fluid absorption while, in the zone of DBP from 100 to 30 mmHg, there is secretion with the peak secretion at 60 mmHg. The agreement between the calculated curve with the data points transferred from Figure2 is remarkably good (*R*^2^ = 68%*, P* *<* 0.001) and goes some way to supporting the plausibility of the proposed process for intestinal fluid secretion.

### Relevance to secretory diarrhea

The applicability of the present results which indicate a role for filtration of fluid from the mucosal capillary bed as a consequence of increased capillary pressure needs to be considered in the context of toxins reported to cause secretory diarrhea.

**Figure 7:**
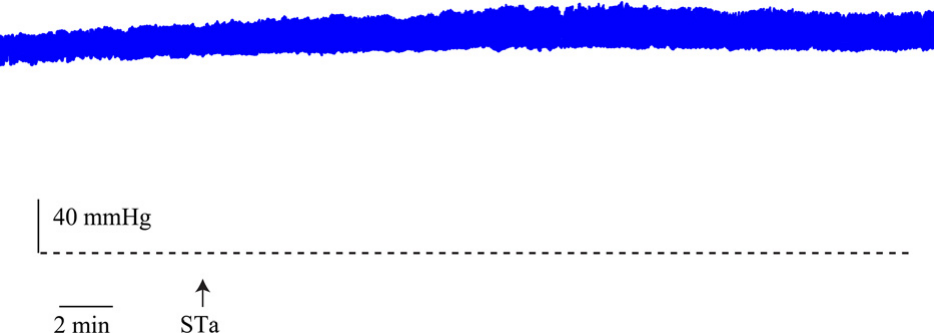
Effect of addition of 40 ng mL^−1^ STa toxin to jejunal Krebs-glucose perfusate (STa) on ABP. The dashed horizontal line denotes 0 mmHg.

Previous results in vivo have indicated that, while *E*. *coli* STa toxin reduced absorption, it did not promote active secretion of fluid into the jejunum (Sach et al. 1976; Rolfe and Levin 1994; Lucas et al. 2005, 2008, 2011). This result was confirmed in additional studies on three animals in which absorption of fluid from a Krebs-glucose perfusate was reduced from −89 to +1.9 *μ*L cm^−1^ h^−1^. It was also clear that perfusion of the jejunum with STa toxin was without effect on the DBP which was shown in five experiments (*P* = 0.29) (Fig.7), which is in marked contrast to the effects of *V*. *cholerae* toxin. When this was administered to conscious/lightly anesthetized dogs, there was fluid loss from the rectum of one liter over the first 5 h, which was preceded by a decline in mean ABP from 100 mmHg down to 30 mmHg and by an increase in superior mesenteric blood flow (Carpenter et al. 1969). A similar result has also been reported in anesthetized cats with the additional finding that the reduced ABP and increased mucosal blood flow may arise by stimulation of VIP release from intestinal neurones by the cholera toxin (Cassuto et al. 1981). At the histological level, increased blood flow was also evident after exposure to *V*. *cholerae* toxin, as shown by villi which were congested with edema and blood which had entered through increased gaps between capillary endothelial cells (Patnaik and Ghosh 1966; Lee 1977) and by distended mucosal vasculature and lymphatic vessels (Norris et al. 1967). Further evidence relating to the importance of blood flow in cholera-induced secretion comes from the experiments of Strombeck (1972) who induced intestinal secretions with *V*. *cholerae* toxin in vivo; however, once the intestinal loops had been excised and mounted in vitro, the secretions abated and normal absorption ensued. Hence, these results demonstrate that secretion is only convincingly detected in the presence of an intact vasculature and appropriate pressure gradient. The extent to which these results from studies on animal models can be related to the human condition is, however, restricted by the dearth of human ABP data and the fact that fluid loss *per se* is treated by oral and I.V. fluid transfusions. There are studies on human volunteers in whom ABP was reported to remain constant though the toxin was delivered in small amounts into a restricted length of jejunum (Petritsch et al. 1992). By contrast, hospital patients with acute phase cholera are always reported to be markedly hypotensive (Rogers 1909; Carpenter et al. 1961) with the ABP as low as 54 mmHg (Banwell et al. 1970). It is noteworthy that newly admitted patients who received three times the normal I.V. infusion made a remarkable recovery compared with those who received the standard infusion (Carpenter et al. 1961), though whether intestinal fluid loss had abated was not reported. On balance, the results point toward the importance of ABP in cholera-induced secretory diarrhea and the basis for this may reside in the increased capillary filtration arising from increased capillary pressure. Whether this also holds for secretory diarrhea caused by the *E*. *coli* toxins remains an open question, though the absence of an action of *E*. *coli* STa toxin on ABP in animal experiments would account for the absence of net secretion in these experiments.

In conclusion, our results strongly suggest that at least a major component of the secretory response in secretory diarrhea has a vascular origin due to the systemic effect of *V*. *cholerae* toxin causing systemic vasodilatation and consequently increasing mucosal capillary pressure to cause exudation of fluid. However, there has to be a recognition that our conclusion is based on inferences about capillary pressure which remains to be measured directly during exposure to *V*. *cholerae* toxin and that our results pertain to the rat model, whereas validation in a large animal model would be desirable. While methodologically, this will require further technically demanding experiments, there is a parallel direction in which work could proceed: this would be to explore the efficacy of administration of a specific vasoconstrictor agent devoid of cardiac actions to test the effects of restoration of the ABP during exposure to *V*. *cholerae* toxin. While we have already demonstrated that vasopressin I.V. promotes absorption (Fig.2), there is a more specific vasopressor agent such as felypressin, a synthetic analog of vasopressin, which acts more specifically on the vasculature and has a reduced antidiuretic action (Martindale, 2011). This and other vasopressin analogs may be a future avenue for a possible therapeutic intervention in the clinical management of secretory diarrhea.

## Appendix

*P* and *π* are the hydrostatic and oncotic pressures, respectively, in the capillary (*c*), interstitial fluid (*i*), and luminal (*l*) compartments. *J*_ci_ is the flux from capillaries to interstitial fluid, *J*_il_ is the flux from interstitial fluid to lumen, *K*_ci_ is the filtration coefficient between the capillaries and interstitial fluid, and *K*_il_ is the filtration coefficient between the interstitial fluid and lumen. The value of *K*_ci_ obtained for cat mesenteric capillaries of 125 *μ*L h^−1^ mmHg^−1^ g^−1^ (Granger et al. 1979) far exceeds the value of *K*_il_ of 9.4 *μ*L h^−1^ mmHg^−1^ g^−1^ obtained as the negative slope in Figure2, converted to units per gram weight. The reflection coefficients (*σ*) have been set to unity (Granger et al. 1979). The fluxes between the compartments are as follows (Taylor 1981):

Since *P*_*l*_ & *π*_*i*_ are zero

At steady state

Since

Thus, the flow of fluid from capillaries into the lumen is mainly determined by the capillary hydrostatic and oncotic pressure difference and the filtration coefficient between the interstitial fluid and lumen.
